# Effect of Oral carnosine supplementation on urinary TGF-β in diabetic nephropathy: a randomized controlled trial

**DOI:** 10.1186/s12882-021-02434-7

**Published:** 2021-06-26

**Authors:** Narongrit Siriwattanasit, Bancha Satirapoj, Ouppatham Supasyndh

**Affiliations:** Department of Medicine, Division of Nephrology, Phramongkutklao Hospital and College of Medicine, Bangkok, 10400 Thailand

**Keywords:** Carnosine, Diabetic nephropathy, Urinary TGF-β

## Abstract

**Background:**

Activation of the transforming growth factor beta (TGF-β) pathway is a significant contributor to the pathogenesis of diabetic nephropathy. Carnosine is a dipeptide that can inhibit TGF-β synthesis. We tested the hypothesis that carnosine supplement added to standard therapy will result in reduced urinary TGF-β levels among patients with diabetic nephropathy.

**Methods:**

We randomly assigned 40 patients with diabetic nephropathy and albuminuria 30–299 mg/day to treatment with carnosine (2 g/day) or placebo for 12 weeks. Urinary TGF-β level was determined using ELISA, urine albumin was ascertained by immunonephelometric assay, and renal function and metabolic profiles were determined at baseline and during 12 weeks of active treatment. Primary outcome was decrease in urinary levels of TGF-β.

**Results:**

The 2 groups were comparable for baseline characteristics, blood pressure, urine albumin, urine TGF-β and renal function measurements. Urinary TGF-β significantly decreased with carnosine supplement (− 17.8% of the baseline values), whereas it tended to increase with placebo (+ 16.9% of the baseline values) (between-group difference *P* < 0.05). However, blood urea nitrogen, serum creatinine, glomerular filtration rate and other biochemical parameters remained unchanged during the study period including urinary albuminuria. Both groups were well tolerated with no serious side-effects.

**Conclusions:**

These data indicated an additional renoprotective effect of oral supplementation with carnosine to decrease urinary TGF-β level that serves as a marker of renal injury in diabetic nephropathy.

**Trial registration:**

Thai Clinical Trials, TCTR20200724002. Retrospectively Registered 24 July 2020.

## Background

Diabetic nephropathy is the leading cause of chronic kidney disease (CKD), and the foremost cause of end stage renal disease (ESRD) [1]. The standard treatment for diabetic nephropathy includes controlling glycemia and blood pressure and reducing albumin leakage in urine using angiotensin converting enzyme inhibitors (ACEIs) or angiotensin receptor blockers (ARBs) [2]. These can reduce the number of patients receiving renal replacement therapy which eventually reduces cost of treatment for patients with diabetic nephropathy.

Hyperglycemia induces an abnormal activation of glucose-dependent pathways. i.e., the polyol pathway, hexosamine pathway and protein kinase C pathway in producing multiple substances, including transforming growth factor beta (TGF-β), vascular endothelial growth factor (VEGF), interleukine-1 (IL-1), interleukine-6 (IL-6) and tissue necrosis factor (TNF) [3, 4]. Increased urinary TGF-β level among patients with diabetes stimulates the canonical pathway (ALK 5, Smad 2/3) and alternate pathway (ALK 1, Smad 1/5) [5]. The activation of the canonical pathway induces extracellular matrix accumulation at the glomerular basement membrane (GBM) and mesangium. In addition, the activation of the alternate pathway induces podocyte injury causing foot process effacement. Therefore, TGF-β and activation of the metabolic pathway are important factors in developing diabetic nephropathy [6]. Treatment to reduce TGF-β level in the urine may be able to slow the deterioration of diabetic nephropathy [7].

Carnosine is an amino acid found in nature, synthesized from L-histidine and beta-alanine (carnosine synthase) and degraded by the enzyme carnosinase [8]. Carnosine has many biological qualities that can slow CKD progression and prevent diabetic nephropathy from developing [9, 10]. One of the proposed mechanisms is that it inhibits the synthesis of TGF-β [11]. It has been hypothesized that individuals with two copies of the CNDP1 Mannheim have lower activity of plasma carnosinase, leading to higher plasma carnosine concentrations and a lower risk of diabetic nephropathy [11]. One study has shown that oral carnosine supplementation could reduce albuminuria and urinary alpha-1 microglobulin level in type 1 diabetes [12]. Presently, no studies have yet been conducted among adult type 2 diabetes mellitus (T2DM) patients with diabetic nephropathy. The study aimed to assess the effect of oral carnosine supplementation on levels or urinary TGF-β and albumin in patients with T2DM.

## Methods

**Fig. 1:**
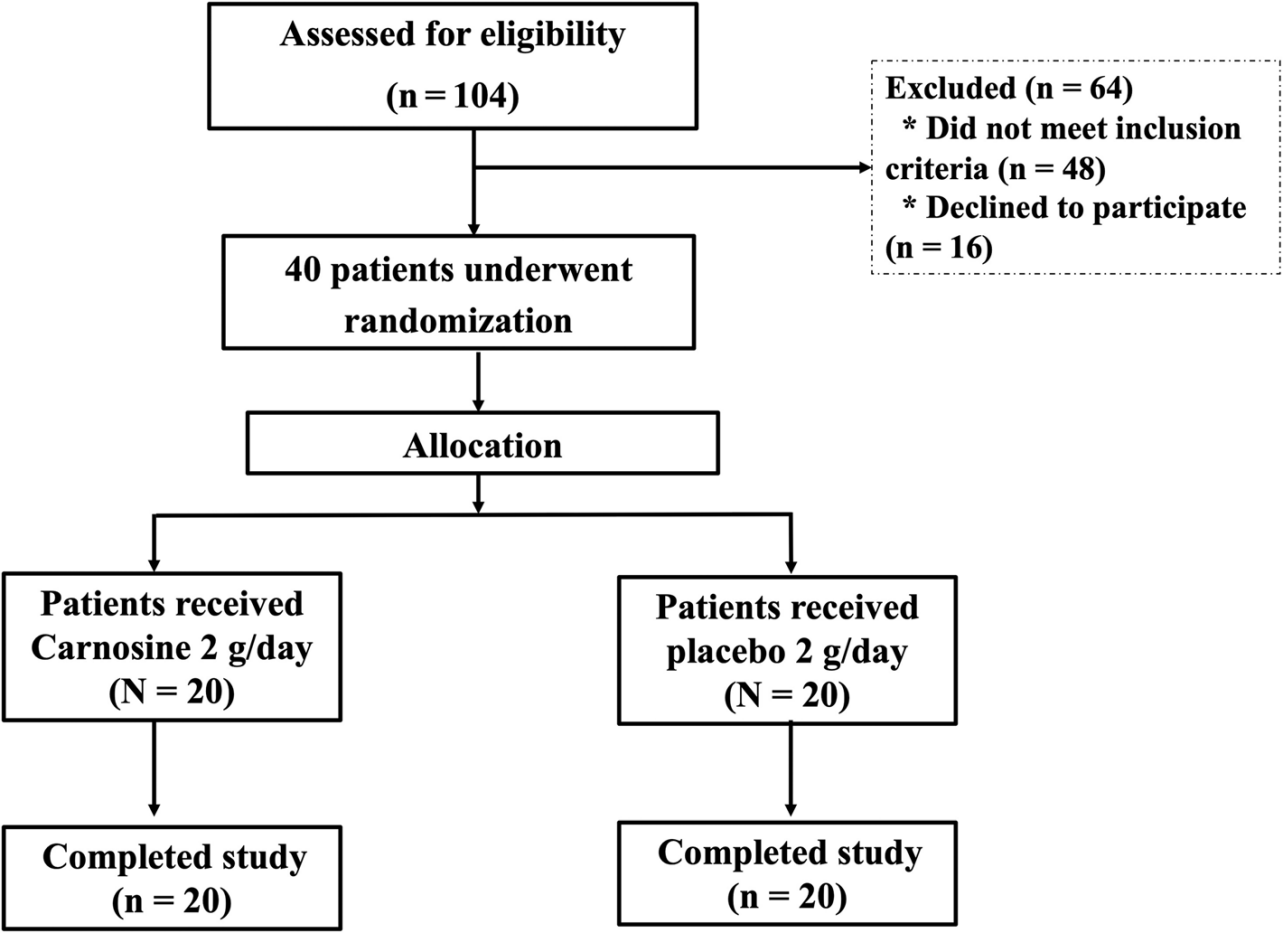
Flow chart of study

The study was a double-blind randomized controlled trial, comparing carnosine supplementation with placebo, alongside conventional treatment. The study was conducted among patients with T2DM treated at Phramongkutklao Hospital between 1 April 2018 and 31 March 2019, with all subjects selected by inclusion criteria. There was assistant researcher who recruited and enrolled participants in this study. Drug administration was according to a predetermined schedule generated from block of four random numbers in a 1:1 ratio based on a computer-generated randomization sequence maintained within the investigational drug pharmacy with allocation concealment by opaque sequentially numbered sealed envelope. As shown in Fig. 1. Group 1 supplemented 2 g/day oral carnosine (Tokai Bussan CO., LTD, Tokyo, Japan.) in gelatin capsules for 12 weeks. The dose was split into 2 × 500 mg taken after breakfast and dinner. Group 2 followed the same schedule but consumed a matched placebo containing starch. The participants had to take 2 capsules each time, after breakfast and dinner for 12 weeks, the same as group 1. The study complies with the Declaration of Helsinki (1964). The study was registered at Thai Clinical Trials Registry (TCTR) (TCTR20200724002).

The inclusion criteria included T2DM with diabetic nephropathy according to KDOQI Guidelines and Chronic Kidney Disease 2007 criteria [13], age of 18 years old or older, urine albumin-creatinine ratio (UACR) of 30 to 299 mg/g creatinine (Cr) at least two in three within three to 6 months, stable dose of ACEIs or ARBs for blood pressure control at least 3 months before enrolling, and stable hemoglobinA1C (HbA1C) within 3 months before the study. The exclusion criteria comprised active infections, CKD from nondiabetic cause, advanced malignancy, history of hypersensitivity to protein nutrients, problems with nutrient absorption of the gastro-intestinal tract and liver disease.

The data we collected before and after in this study, were relevant information on diabetic nephropathy, including diagnostic criteria, duration of the disease and complications of diabetes mellitus such as diabetic retinopathy and diabetic neuropathy. Also, other underlying diseases, including hypertension, heart disease, liver disease, infectious diseases and malignancy were recorded. The history of medication including antihypertensive drugs and lipid lowering agents were recorded. Physical examination data including height, weight, blood pressure and body mass index (BMI) were collected. All subjects fasted for at least 12 h overnight before all blood drawing. The laboratory tests including fasting plasma glucose (FPG), HbA1C, blood urea nitrogen, Cr, calculation of estimated glomerular infiltration rate using the 2009 Chronic Kidney Disease Epidemiology Collaboration Equation, total cholesterol, triglyceride, low density lipoprotein and high-density lipoprotein were noted.

Thirty milliliters of fresh urine were centrifuged at 4000 rpm for 10 min, then stored at − 80 °C until assayed. Urinary TGF-β level were tested by enzyme-linked immunosorbent assay (IBL-America, Inc. Minneapolis, MN). All specimens were diluted often to obtain concentration at the optimal density according to the ELISA kit instruction. Coefficients of variation for urine tubular biomarkers assays were < 10%, for intra-assay and inter-assay variation. UACR by immunonephelometric assay method, before and after receiving carnosine or placebo for a period of 12 weeks is shown in Fig. 1.

### Follow-up study results

The researcher verified consistent carnosine intake by asking for the remaining tablets and followed up the side effects of carnosine intake by using the adverse effects assessment form (Naranjo’s algorithm) [14]. Data of adherence to oral carnosine intake was recorded. The primary outcome was the change of urinary TGF-β level after 12 weeks in the oral carnosine supplementation group, compared with that of the placebo group. The secondary outcome was the improving level of UACR after 12 weeks in the oral carnosine supplementation group compared with that of the placebo group.

### Statistical analysis

Data were analyzed using the commercially available SPSS 22.0 statistical software program (SPSS, Chicago, IL, US). Descriptive statistics were used to present general information, laboratory results and urinary substances measurement level, including percentages, averages, and standard deviations in the case of normal distributed continuous data. Inferential statistics was used to compare between general information, laboratory results and the percentage changes of variables in the oral carnosine supplementation and placebo groups, based on Student’s test statistics. Pearson chi-square test or Fisher’s exact test was used for discrete or categorical variables. Two-way analysis of variance (ANOVA) with repeated measures and paired-sample t tests was used for the continuous variables and presented by the relative risk of 95% confidence intervals with *p*-value less than 0.05, regarded as statistically significant.

### Results

From the screening, of a total of 104 patients with T2DM and nephropathy, 64 were excluded. The included 40 patients were randomly divided in two groups and all of them were 100% adherent to the carnosine or placebo prescription based on pill counts. Baseline laboratory tests and metabolic profiles were found between the two groups as shown in Table 1.

**Table 1:** Baseline characteristics of patients

Variables	Placebo (*N*=20)	Carnosine (*N*=20)	*P* value
Male, n (%)	4 (20.0)	11 (55.0)	0.022
Age (years)	57.0±6.9	55.6±4.8	0.463
Duration (years)	13.0±8.8	10.5±6.5	0.323
Body weight (kg)	73.2±15.3	81.6±18.5	0.127
Body mass index (kg/m^2^)	28.4±5.2	30.3± 5.6	0.284
Systolic blood pressure (mmHg)	134.2±16.4	134.1±11.8	0.965
Diastolic blood pressure (mmHg)	78.2±7.0	78.6±11.5	0.895
Comorbid diseases (N, %)			
Hypertension	14 (70.0)	17 (85.0)	0.451
Dyslipidemia	17 (85.0)	16 (80.0)	1.000
Coronary heart disease	1 (5.0)	-	1.000
Chronic lung disease	-	1 (5.0)	1.000
Anti-hypertensive drugs (N, %)			
ACEI	1 (5.0)	3 (15.0)	0.605
ARB	11 (55.0)	12 (60.0)	0.749
CCB	9 (45.0)	13 (65.0)	0.204
Thiazide	3 (15.0)	1 (5.0)	0.605
Hydralazine	1 (5.0)	-	1.000
Doxazocin	2 (10.0)	3 (15.0)	1.000
Anti-glycemic drugs (N, %)			
Metformin	15 (75.0)	20 (100.0)	0.047
Sulfonylurea	7 (35.0)	5 (25.0)	0.490
Thiazolidinedione	5 (25.0)	9 (45.0)	0.185
DPP4-inhibitor	7 (35.0)	5 (25.0)	0.490
SGLT-2 inhibitor	7 (35.0)	2 (10.0)	0.127
GLP-1 agonist	1 (5.0)	2 (10.0)	1.000
Laboratory parameters			
FPG (mg/dL)	131.0±30.3	168.6±52.7	0.009
HemoglobinA1C (%)	7.8±1.8	7.8±1.5	0.971
Triglycerides (mg/dL)	159.4±88.8	157.5±112.9	0.953
Cholesterol (mg/dL)	156.6±37.6	157.8±29.9	0.913
LDL-cholesterol (mg/dL)	92.3±35.8	114.9±81.1	0.080
HDL-cholesterol (mg/dL)	50.9±2.6	49.4±2.3	0.262
BUN (mg/dL)	17.1±7.2	15.6±5.7	0.456
Creatinine (mg/dL)	0.9±0.3	0.9±0.2	0.677
GFR (mL/min/1.73 m^2^)	78.6±22.1	81.6±19.7	0.655
Urine TGF-β (pg/mgCr)	82.9±57.1	89.1±75.9	0.775
UACR (mg/gCr)	114.7±64.8	114.8±56.4	0.997

Data in the table are shown with average + standard deviation and percentages.

*ACEI* angiotensin converting enzyme inhibitor, *ARB* angiotensin receptor blockade, *BUN* blood urea nitrogen, *CCB* Calcium channel blocker, *DPP4-inhibitor* dipeptidyl peptidase-4 inhibitor, *SGLT-2 inhibitor* sodium glucose co-transporter inhibitor, *GLP-1 agonist* glucagon-like peptide-1 receptor agonist, *GFR* estimated glomerular filtration rate, *LDL* low density lipoprotein cholesterol, *HDL* high density lipoprotein cholesterol

### Change of urine TGF-β after treatment

**Fig. 2:**
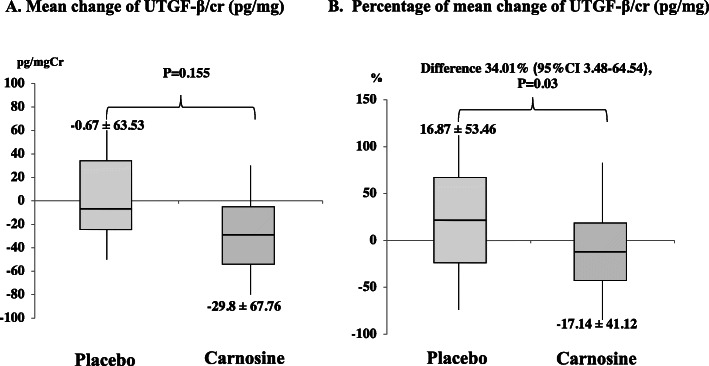
Change of urine TGF-β after treatment. Box-and-whisker-plot diagrams show the (**A**) mean change of urine TGF-β (pg/mgCr) and (**B**) percentage of mean change of urine TGF-β (pg/mgCr) after 12 weeks of taking carnosine

After 12 weeks, no significant differences were found on mean change of urine TGF-β between the two groups as shown in Fig. 2A. After additional analysis, the oral carnosine supplement group had a decreased percentage of mean change of urine TGF-β Cr ratio from baseline by 17.14%. Whereas the percentage of placebo increased by 16.87%. Both groups had a percent mean difference of 34.01 and differed significantly (*P* = 0.03, 95% CI 3.48 to 64.54), as shown in Fig. 2B.

### Change of urine albumin after treatment

**Fig. 3:**
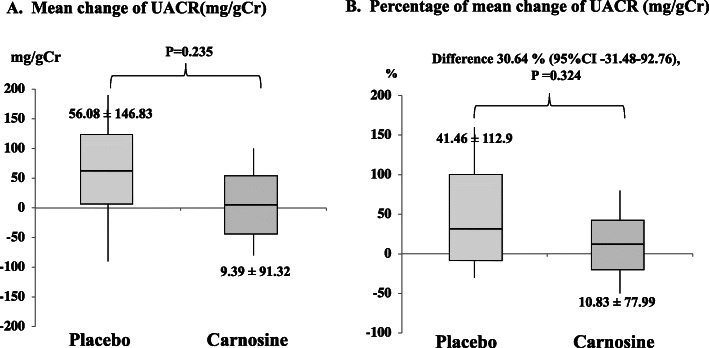
Change of urine albumin after treatment. Box-and-whisker-plot diagram shows the (**A**) mean change of UACR and (**B**) percentage of mean change of UACR (mg/gCr) after 12 weeks of taking carnosine

The percentage of mean change of UACR increased from baseline by 10.83% (mean ± SD = 10.83 ± 77.99 mg/gCr) in the carnosine group. However, in the placebo group, the percentage of mean change of UACR increased by 41.46% (mean ± SD = 41.46 ± 112.9 mg/gCr). Both groups exhibited a percent mean difference of 30.64%, without significance (*P* = 0.324, 95% CI − 31.48 to 92.76) and did not differ significantly concerning mean change of urine albuminuria as shown in Fig. 3A and B.

### Metabolic profiles and adverse events after treatment

After 12 weeks, BMI, blood pressure, renal function, HbA1C and lipid profiles of all patients remained unchanged from baseline, as shown in Table 2 and Table 3. Our participants did not experience any side effects of carnosine during the study.

**Table 2:** Change of variables after 12 weeks of treatment

Variables	Placebo (*N*=20)	Carnosine (*N*=20)	*P* value
Body weight (kg)	-0.65±1.64	-0.22±2.23	0.167
Body mass index (kg/m^2^)	-0.25±0.65	-0.18±0.97	0.101
Systolic blood pressure (mmHg)	-0.90±7.38	-3.15±12.40	0.217
Diastolic blood pressure (mmHg)	-1.95±6.20	0.35±6.32	0.424
BUN (mg/dL)	1.76±5.23	0.48±5.59	0.199
Creatinine (mg/dL)	0.01±0.11	-0.03±0.13	0.523
GFR (mL/min/1.73m^2^)	-0.29±8.04	4.18±11.82	0.232
Fasting plasma glucose (mg/dL)	-25.9 ± 73.6	-16.7 ± 73.59	0.696
HemoglobinA1C (%)	-0.07±1.46	-0.33±0.78	0.486
LDL (mg/dL)	10.30±47.40	-23.40±86.60	0.556
HDL (mg/dL)	1.60±12.10	-1.90±11.0	0.929
Cholesterol (mg/dL)	9.84±51.90	-10.10±41.60	0.984
Triglyceride (mg/dL)	-9.20±70.70	-0.50±63.70	0.653

Data in the table are shown with average + standard deviation.

*BUN* blood urea nitrogen, *GF* glomerular filtration rate, *LDL* low density lipoprotein cholesterol, *HDL* high density lipoprotein cholesterol

**Table 3:** Mean variables at 12 weeks of treatment

Variables	Placebo (*N*=20)	Carnosine (*N*=20)	*P* value
Body weight (kg)	72.54±14.7	81.34±18.9	0.117
Body mass index (kg/m^2^)	28.18±5.0	30.12±5.6	0.268
Systolic blood pressure (mmHg)	133.35±16.5	130.9±13.6	0.611
Diastolic blood pressure (mmHg)	76.2±7.9	78.9±11.7	0.763
BUN (mg/dL)	18.89±5.6	16.06±5.9	0.217
Creatinine (mg/dL)	0.895±0.03	0.896±0.3	0.832
GFR (mL/min/1.73m^2^)	78.34±23.6	85.78±20.2	0.434
Fasting plasma glucose (mg/dL)	156.85±70.2	151.9±47.5	0.171
HemoglobinA1C (%)	7.86±1.8	7.48±1.3	0.716
LDL (mg/dL)	102.59±48.1	91.5±35.2	0.656
HDL (mg/dL)	51.17±13.2	46.89±9.2	0.404
Cholesterol (mg/dL)	166.43±51.6	147.6±35.1	0.383
Triglyceride (mg/dL)	150.22±77.1	157.02±71.7	0.926

Data in the table are shown with average + standard deviation.

*BUN* blood urea nitrogen, *GF* glomerular filtration rate, *LDL* low density lipoprotein cholesterol, *HDL* high density lipoprotein cholesterol

## Discussion

This study was the first randomized controlled trial showing the statistically significant differences in the data regarding oral carnosine supplementation among patients with T2DM and nephropathy, to reduced urinary TGF-β compared with placebo. This was consistent with related research investigating patients with type 1 diabetes and nephropathy. Elbarbary et al. reported that carnosine could reduce urine alpha-1 microglobulin, which is a urine biomarker of glomerular and tubular injury among diabetic patients, as well as urinary TGF-β [12]. Several studies in vitro studies and animal models also demonstrated anti-apoptosis, anti-inflammatory, anti-oxidant, antiglycation, antiproteinuric and vasculoprotective effects of carnosine [15–18].

Reduced urinary TGF-β is a biomarker for CKD progression [7]. It has been shown that carnosine may have a reno-protective effect on ischemia/reperfusion-induced acute kidney injury in animal models [19] and attenuates the development of patients with T2DM and nephropathy [20]. Whereas we found that oral carnosine supplementation did not reduce urine albumin, which differed from the study of Elbarbary et al. [12]. The finding might be explained in that baseline patients’ conditions in this study were more severe regarding the degree of diabetic nephropathy. Higher age, urine albumin and comorbid illness including hypertension, dyslipidemia, and obesity were observed in our study. On the contrary, the subjects in related studies had shorter duration of diabetes without history of underlying diseases reported. Our study found that early biomarkers of kidney injury including urine TGF-β level was lower in the carnosine group. Thus, a follow-up of longer duration might show significantly decreased levels of urine albumin.

Additional renal benefits of carnosine treatment were improved glycemic and metabolic control [21, 22]. An in vivo study in diabetes-induced mice receiving carnosine supplements showed reduced FPG levels, decreased insulin resistance and increased β-cell mass [23–25]. In addition, a study of Elbarbary et al., investigated among children with type 1 diabetes mellitus found that oral carnosine supplementation for 12 weeks could significantly reduce HbA1C compared with placebo [12], which differed from our study. This was due to the difference in baseline HbA1C where average baseline HbA1C levels were 7.8% in our study and 8.2% in previous study [12]. Patients with T2DM in our study were already able to effectively control their HbA1C levels at 7.8%, as we could not see any additional benefit of carnosine on reducing HbA1C level. Another in vitro study of Lee YT, et al. showed that carnosine could improve lipid metabolism [26]. Moreover, carnosine could reduce lipid peroxidation, atherogenic ApoB lipoproteins and triglycerides in plaques of mice [27]. The study among children with type 1 diabetes found that receiving carnosine for 12 weeks could improve cholesterol level [12]. The lipid outcome was undetected in our study, because approximately 80% of our adult subjects received strong lipid lowering agents.

The limitation of our study was that we did not evaluate major renal outcomes including ESRD, double serum Cr and dialysis. The main outcome was only biomarkers of kidney progression including urine TGF-β and albumin. Due to the short duration, our study could not conclude any long-term effects of carnosine on urine TGF-β reduction and renal outcomes. Therefore, the long-term side effects of carnosine are needed to be further investigated.

## Conclusion

In summary, the study showed that oral carnosine supplementation could reduce urinary TGF-β level in T2DM with diabetic nephropathy, but without significant effects on urine albumin, indicating an additional renoprotective effect from conventional therapy. Further study is needed to determine the long-term effects of oral carnosine supplementation on delayed renal progression in T2DM as a result of the decreased level of urinary TGF-β.

## Data Availability

The excel of individual clinical data used to support the findings of this study are available from the corresponding author upon request.

## Abbreviations

ACEIs: Angiotensin converting enzyme inhibitors
ARBs: Angiotensin receptor blockers
BMI: Body mass index
CKD: Chronic kidney disease
Cr: Creatinine
ESRD: End stage renal disease
FPG: Fasting plasma glucose
HbA1C: Hemoglobina1c
GBM: Glomerular basement membrane
GLP-1 agonist: Glucagon-like peptide-1 receptor agonist
IL-1: Interleukine-1
IL-6: Interleukine-6
SGLT-2inhibitor: Sodium glucose cotransporter-2 inhibitor
TGF-β: Transforming growth factor beta
T2DM: Type 2 diabetes mellitus
VEGF: Vascular endothelial growth factor
TNF: Tissue necrosis factor
UACR: Urine albumin creatinine ratio

## References

[1] Kim KS, Park SW, Cho YW, Kim SK (2018). Higher prevalence and progression rate of chronic kidney disease in elderly patients with type 2 diabetes mellitus. Diabetes Metab J.

[2] Satirapoj B, Adler SG (2015). Prevalence and Management of Diabetic Nephropathy in Western countries. Kidney Dis (Basel).

[3] Schena FP, Gesualdo L (2005). Pathogenetic mechanisms of diabetic nephropathy. J Am Soc Nephrol.

[4] Satirapoj B, Adler SG (2014). Comprehensive approach to diabetic nephropathy. Kidney Res Clin Pract.

[5] Zheng X, Bhalla V (2015). The missing link: studying the alternative TGF-beta pathway provides a unifying theory for different components of diabetic nephropathy. Diabetes.

[6] Chang AS, Hathaway CK, Smithies O, Kakoki M (2016). Transforming growth factor-beta1 and diabetic nephropathy. Am J Physiol Renal Physiol.

[7] Qiao YC, Chen YL, Pan YH, Ling W, Tian F, Zhang XX, Zhao HL (2017). Changes of transforming growth factor beta 1 in patients with type 2 diabetes and diabetic nephropathy: a PRISMA-compliant systematic review and meta-analysis. Medicine (Baltimore).

[8] Hobart LJ, Seibel I, Yeargans GS, Seidler NW (2004). Anti-crosslinking properties of carnosine: significance of histidine. Life Sci.

[9] Reddy VP, Garrett MR, Perry G, Smith MA (2005). Carnosine: a versatile antioxidant and antiglycating agent. Sci Aging Knowl Environ.

[10] Boldyrev AA, Aldini G, Derave W (2013). Physiology and pathophysiology of carnosine. Physiol Rev.

[11] Janssen B, Hohenadel D, Brinkkoetter P, Peters V, Rind N, Fischer C, Rychlik I, Cerna M, Romzova M, de Heer E, Baelde H, Bakker SJL, Zirie M, Rondeau E, Mathieson P, Saleem MA, Meyer J, Koppel H, Sauerhoefer S, Bartram CR, Nawroth P, Hammes HP, Yard BA, Zschocke J, van der Woude FJ (2005). Carnosine as a protective factor in diabetic nephropathy: association with a leucine repeat of the carnosinase gene CNDP1. Diabetes.

[12] Elbarbary NS, Ismail EAR, El-Naggar AR, Hamouda MH, El-Hamamsy M (2018). The effect of 12 weeks carnosine supplementation on renal functional integrity and oxidative stress in pediatric patients with diabetic nephropathy: a randomized placebo-controlled trial. Pediatr Diabetes.

[13] Saunders W: KDOQI clinical practice guidelines and clinical practice recommendations for diabetes and chronic kidney disease. 2007. 10.1053/j.ajkd.2006.12.005 17276798

[14] Naranjo CA, Busto U, Sellers EM, Sandor P, Ruiz I, Roberts E, Janecek E, Domecq C, Greenblatt D (1981). A method for estimating the probability of adverse drug reactions. Clin Pharm Ther.

[15] Peters V, Zschocke J, Schmitt CP (2018). Carnosinase, diabetes mellitus and the potential relevance of carnosinase deficiency. J Inherit Metab Dis.

[16] Peters V, Yard B, Schmitt CP (2020). Carnosine and diabetic nephropathy. Curr Med Chem.

[17] Liu XQ, Jiang L, Lei L, Nie ZY, Zhu W, Wang S, Zeng HX, Zhang SQ, Zhang Q, Yard B, Wu YG (2020). Carnosine alleviates diabetic nephropathy by targeting GNMT, a key enzyme mediating renal inflammation and fibrosis. Clin Sci (Lond).

[18] Zhao K, Li Y, Wang Z, Han N, Wang Y (2019). Carnosine protects mouse podocytes from high glucose induced apoptosis through PI3K/AKT and Nrf2 pathways. Biomed Res Int.

[19] Kurata H, Fujii T, Tsutsui H, Katayama T, Ohkita M, Takaoka M, Tsuruoka N, Kiso Y, Ohno Y, Fujisawa Y, Shokoji T, Nishiyama A, Abe Y, Matsumura Y (2006). Renoprotective effects of l-carnosine on ischemia/reperfusion-induced renal injury in rats. J Pharmacol Exp Ther.

[20] Albrecht T, Schilperoort M, Zhang S, Braun JD, Qiu J, Rodriguez A, Pastene DO, Kramer BK, Koppel H, Baelde H (2017). Carnosine attenuates the development of both type 2 diabetes and diabetic nephropathy in BTBR Ob/Ob mice. Sci Rep.

[21] Karkabounas S, Papadopoulos N, Anastasiadou C, Gubili C, Peschos D, Daskalou T, Fikioris N, Simos YV, Kontargiris E, Gianakopoulos X, Ragos V, Chatzidimitriou M (2018). Effects of alpha-lipoic acid, carnosine, and thiamine supplementation in obese patients with type 2 diabetes mellitus: a randomized, double blind study. J Med Food.

[22] Kilis-Pstrusinska K (2020). Carnosine and kidney diseases: what we currently know?. Curr Med Chem.

[23] Sauerhofer S, Yuan G, Braun GS, Deinzer M, Neumaier M, Gretz N, Floege J, Kriz W, van der Woude F, Moeller MJ (2007). L-carnosine, a substrate of carnosinase-1, influences glucose metabolism. Diabetes.

[24] Aldini G, Orioli M, Rossoni G, Savi F, Braidotti P, Vistoli G, Yeum KJ, Negrisoli G, Carini M (2011). The carbonyl scavenger carnosine ameliorates dyslipidaemia and renal function in Zucker obese rats. J Cell Mol Med.

[25] Soliman KM, Abdul-Hamid M, Othman AI (2007). Effect of carnosine on gentamicin-induced nephrotoxicity. Med Sci Monit.

[26] Lee YT, Hsu CC, Lin MH, Liu KS, Yin MC (2005). Histidine and carnosine delay diabetic deterioration in mice and protect human low density lipoprotein against oxidation and glycation. Eur J Pharmacol.

[27] Yapislar H, Taskin E (2014). L-carnosine alters some hemorheologic and lipid peroxidation parameters in nephrectomized rats. Med Sci Monit.

